# Exploring organoid and assembloid technologies: a focus on retina and brain

**DOI:** 10.1017/erm.2025.9

**Published:** 2025-03-27

**Authors:** Sara Ouaidat, Alessandro Bellapianta, Franziska Ammer-Pickhardt, Tara Taghipour, Matthias Bolz, Ahmad Salti

**Affiliations:** 1 Research Group Cellular and Molecular Ophthalmology, University Clinic for Ophthalmology and Optometry, Kepler University Hospital, Johannes Kepler University Linz, Linz, Austria; 2 Department of Biosciences & Medical Biology, Paris-Lodron-University of Salzburg (PLUS), Salzburg, Austria

**Keywords:** 3D models, assembloids, axonal projections, brain organoids (BOs), organoids, retinal ganglionic cells (RGCs), retinal organoids (ROs)

## Abstract

**Background:**

The recent emergence of three-dimensional organoids and their utilization as in vitro disease models confirmed the complexities behind organ-specific functions and unravelled the importance of establishing suitable human models for various applications. Also, in light of persistent challenges associated with their use, researchers have been striving to establish more advanced structures (i.e. assembloids) that can help address the limitations presented in the current organoids.

**Methods:**

In this review, we discuss the distinct organoid types that are available to date, with a special focus on retinal and brain organoids, and highlight their importance in disease modelling.

**Results:**

We refer to published research to explore the extent to which retinal and brain organoids can serve as potential alternatives to organ/cell transplants and direct our attention to the topic of photostimulation in retinal organoids. Additionally, we discuss the advantages of incorporating microfluidics and organ-on-a-chip devices for boosting retinal organoid performance. The challenges of organoids leading to the subsequent development of assembloid fusion models are also presented.

**Conclusion:**

In conclusion, organoid technology has laid the foundation for generating upgraded models that not only better replicate in vivo systems but also allow for a deeper comprehension of disease pathophysiology.

## Introduction

In the last decades, animal models and classical two-dimensional (2D) in vitro cultures were particularly used to explore and understand disease pathologies (Refs 1–7). Also, the use of human post mortem tissues was beyond valuable for disease inspection (Refs 8, 9). However, the investigation of disease pathophysiology was rendered challenging due to restricted recapitulation of disease microenvironments that closely mirror in vivo systems, and post mortem tissues represent only the last stage of the disease without insight into its onset and progression. Indeed, this obstacle persisted until the advent of the groundbreaking ‘organoid technology’ (Ref. 10). Organoids, also referred to as ‘mini-organs’, are three-dimensional (3D) in vitro grown structures exhibiting either complete or partial resemblance to in vivo organs in terms of cellular organization, microanatomy and architecture (Ref. 11). These self-assembling miniatures are renowned for their ability to mimic and replicate organ-specific functions.

In fact, organoids can be either generated from pluripotent stem cells, including embryonic stem cells (ESCs) and induced pluripotent stem cells (iPSCs), or multipotent adult stem cells (ASCs) (Refs 12–14). Historically, it all started back in 1907 when Henry Van Peters Wilson revealed the capacity of dissociated sponge cells to differentiate and self-organize into a whole organism (Ref. 15). Later, the regeneration of diverse organs from single-cell suspensions was demonstrated by Weiss’s team, where kidney-, liver- and skin-derived suspensions obtained from chick embryos were able to efficiently reconstitute their respective organs of origin (Ref. 16). Afterwards, a breakthrough in stem cell research was reported following the successful isolation of pluripotent stem cells (PSCs) from mouse embryos (Ref. 17), along with the innovative reprogramming of somatic cells into iPSCs (Ref. 18). In 2006, Kazutoshi Takahashi and Shinya Yamanaka made groundbreaking discoveries in the field of stem cell research. They successfully reprogrammed mouse fibroblast cells to become iPSCs, which have the ability to differentiate into various cell lineages (Ref. 18). In 2012, Shinya Yamanaka was awarded the Nobel Prize in Physiology or Medicine, along with John B. Gurdon, for their discoveries in reprogramming mature cells to become pluripotent. Then, it was not until an experiment conducted by Sato and colleagues in 2009 that the reputation of organoid technology began to flourish, highlighting its immense potential in the fields of regenerative medicine and tissue engineering. In this study, single-leucine–rich repeat-containing G-protein–coupled receptor 5 (Lgr5)–expressing intestinal stem cells were capable of forming 3D organoids, which, in return, self-organized into crypt-villus structures in vitro, in the absence of a non-epithelial cellular niche (Ref. 19). This pivotal research laid the foundation for the further exploration of organoids’ capacities in many other systems.

Ever since their emergence, organoids have been utilized as promising tools in wide arrays of applications such as disease modelling (Refs 20–22), drug screening (Refs 23–25), cell-replacing therapies (Refs 26–28), as well as personalized medicine (Ref. 29). Furthermore, these 3D models have proven to be indispensable in replicating diverse in vivo systems, alleviating persistent difficulties associated with translating findings from animal models to human context (Refs 30, 31). Nevertheless, several challenges remain to be considered upon organoid employment. For example, organoids often display higher susceptibility to cellular stress, hypoxia and necrosis, along with the lack of appropriate vascularization, and most importantly, the absence of inter-regional interactions (Ref. 32). Therefore, several questions are raised: How can 3D in vitro organoid models more accurately reflect human physiology? Can this be accomplished through the development of more advanced structures featuring regional communication and interaction?

The answer to the above-posed questions was later on provided by the establishment of a new model called ‘assembloid’ (Ref. 33). Assembloids can be defined as 3D fusion models obtained by the process of combining two or more different organoid structures (i.e. multi-region assembloids) or through integrating missing cell types within organoids (i.e. multi-lineage assembloids) (Ref. 34). These complex structures have been utilized as alternatives to organoids for modelling inter-cellular as well as inter-regional interactions.

In the present review, we will introduce the distinct organoid types that are currently available in the research field, with a focus on brain (BOs) and retinal organoids (ROs). We will also discuss the application of organoid technology in the field of regenerative medicine (i.e. transplantation) and we will highlight the topic of photostimulation as well as the implementation of microfluidic technology for retinal organoids. Moreover, the diverse assembloid models and their importance in the investigation of axonal projection and pathfinding will be demonstrated.

## Organoids and disease modelling

**Figure 1.**
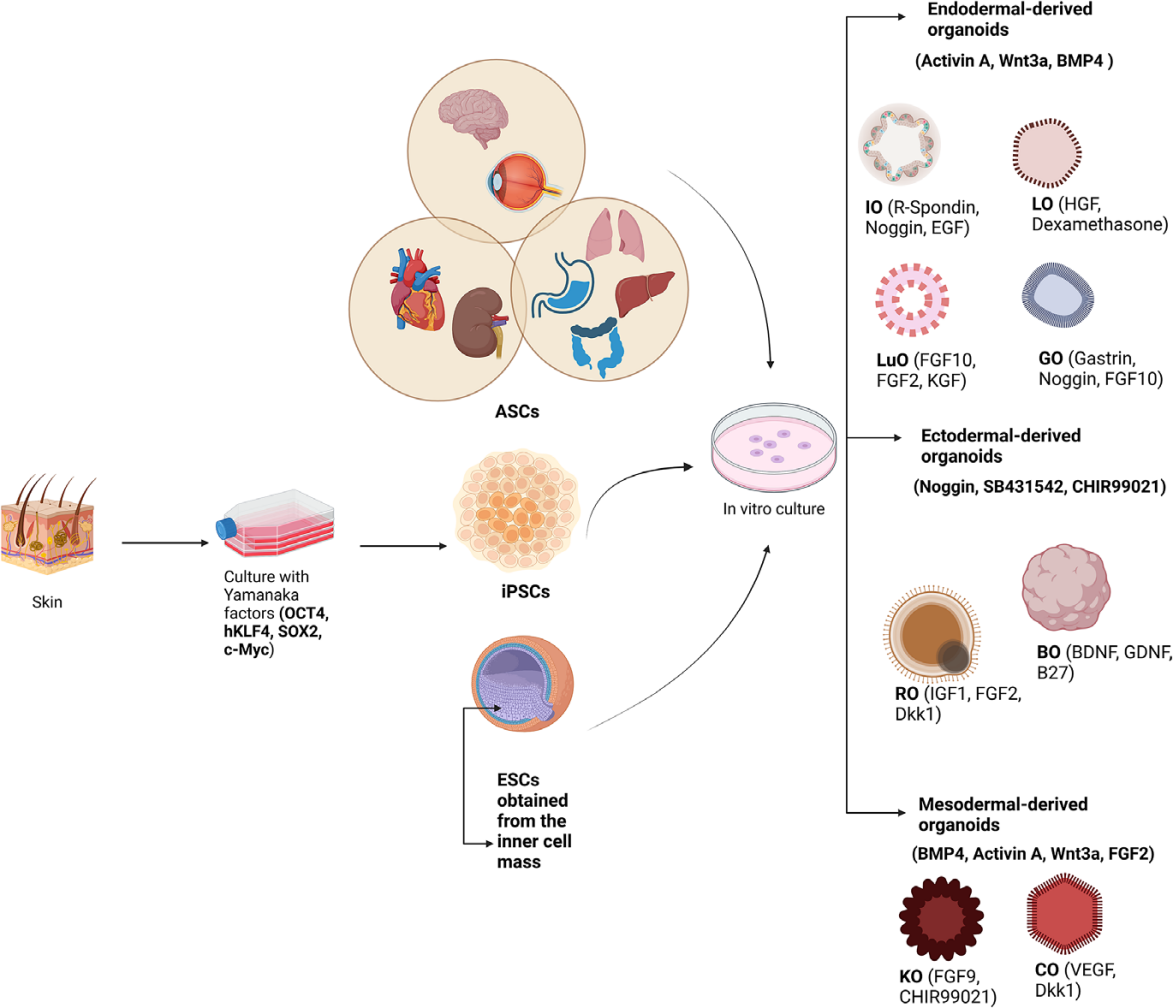
Organoids can be derived from either adult stem cells (ASCs), induced pluripotent stem cells (iPSCs) obtained via genetic reprogramming, or embryonic stem cells (ESCs) derived from the inner cell mass of the blastocyst. General germ layer specification factors are required for the establishment of diverse organoids, including activin A, Wnt3a and BMP4 for endodermal-derived; noggin, SB431542 and CHIR99021 for ectodermal-derived; BMP4, activin A, Wnt3a and FGF2 for mesodermal-derived organoids. In addition, a number of specific factors denoted for lineage specification of each organoid type are presented within brackets. LO: liver organoids; LuO: lung organoids; GO: gastric organoids; IO: intestinal organoids; BO: brain organoids; RO: retinal organoids; KO: kidney organoids; and CO: cardiac organoids. Created with Biorender.

The rapid advancement in the field of organoid technology has enabled researchers to generate different types of these 3D structures depending on the disease and/or organ of interest. Based on the origin of the organ during embryogenesis, ectodermal-, mesodermal- and endodermal-derived organoids were generated. Figure 1 summarizes the most important organoids that are available to date as disease models. These organoids can be generated by applying the specific factors expressed during germ layer formation, and subsequently by using the factors responsible for cell lineage specification.

The organoids derived from mesodermal and endodermal specification have proven their importance as disease models. For instance, CRISPR-mutant kidney organoids and kidney organoids derived from diseased human iPSCs were able to recapitulate the cyst production phenotype, characteristic of polycystic kidney disease (Refs 35, 36). Moreover, patient-derived cardiac organoids proved to be efficient in modelling diseases such as Duchenne muscular dystrophy and genetic cardiomyopathies (Refs 37, 38). Endodermal-derived liver and intestinal organoids, among others, have been also successfully utilized as appropriate models for the investigation of disease pathophysiology such as biliary atresia, Wilson’s disease and cystic fibrosis (Refs 39, 40). For additional information on mesodermal-derived organoids, refer to the following recent reviews concerning kidney (Refs 41–43) and cardiac organoids (Refs 44, 45). Concerning the endodermal-derived ones, refer to the mentioned reviews for intestinal (Refs 46–48), gastric (Ref. 49), liver (Ref. 50) and lung organoid models (Refs 51, 52).

Among the ectodermal-derived models, BOs and ROs have gained great importance owing to their significance in disease modelling due to their cellular diversity and interaction complexity. For instance, Lancaster and colleagues demonstrated the efficacy of cerebral organoids in the recapitulation of certain aspects of cortical development as well as microcephaly (Ref. 53). In this study, patient-derived cerebral organoids exhibited premature neuronal differentiation, potentially elucidating the disease phenotype, as confirmed by an increase in the number of bromodeoxyuridine-/doublecortin-positive (BrdU^+^/DCX^+^) cells within these structures (Ref. 53). In another study, cortical organoids derived from iPSCs of patients with Miller–Dieker syndrome (MDS) (i.e. severe form of lissencephaly) displayed elevated neuroepithelial stem cell apoptosis and increased horizontal divisions of these cells, along with abnormal neuronal migration (Ref. 54). These attributes, indeed, make them crucial tools for simulating neurological disorders like lissencephaly (Ref. 54). Furthermore, Conforti’s team utilized 3D cortical organoids for modelling Huntington’s disease (HD), where a number of genes implicated in neuronal differentiation and migration were found to be downregulated within HD organoids (Ref. 55). Additionally, key characteristics of traumatic brain injury (TBI) including neuronal death, tau phosphorylation and transactive response DNA-binding protein 43 (TDP-43) nuclear egress were successfully replicated via mechanically injured cortical organoids, highlighting their importance in TBI research (Ref. 56). In another study, human midbrain organoids developed from iPSCs harbouring SNCA triplication were found to elicit dopaminergic neuronal loss and aggregations of α-synuclein (Ref. 57), making them essential tools for Parkinson’s disease (PD) investigation (Refs 58–61). It is worth noting that diverse BOs have been also adopted as a means for modelling numerous viral infections such as Zika, SARS-Cov-2, HSV-1, and HIV-1, among others (Ref. 62).

Likewise, the significance of ROs in disease modelling is undeniable (Ref. 1). Indeed, in a study done by Deng and colleagues, retinitis pigmentosa (RP) was recapitulated using patient-specific iPSC-derived 3D ROs (Ref. 63). Interestingly, not only did these ROs demonstrate reduced photoreceptor number but also they featured a decline in retinal gene expression as well as ciliary length (Ref. 63). Furthermore, transcriptomic profiling of ROs derived from patients with PRPF31 mutation showed disrupted pre-mRNA splicing events for cellular adhesion and cilia genes, contributing to RP. In addition, correction of PRPF31 mutation using CRISPR/Cas9 gene editing approach restores protein expression and key RP cellular defects, such as cilia length, providing proof of concept for future gene therapy (Ref. 64). Moreover, RO models, generated from iPSCs of patients carrying USH2A mutation, exhibited decreased laminin expression, impaired retinal layer formation and compromised photoreceptor development, coupled with heightened neuronal apoptosis (Ref. 65). These findings, in fact, highlight their effectiveness in recapitulating pathophysiological features associated with USH2A mutations observed in cases of non-syndromic RP (Ref. 65). Besides, X-linked retinitis pigmentosa (XLRP) was explored using both RP2 knockout (RP2-KO) iPSC- and RP2 patient-derived iPSC-ROs (Ref. 66). As a matter of fact, this study has shed light on the capabilities of ROs in modelling the state of photoreceptor degeneration reported in XLRP, as both models showed maximal photoreceptor cell death by day 150, followed by outer nuclear layer thinning by day 180 of culture (Ref. 66). Furthermore, Zhang *et al.*, through utilizing neural ROs derived from a patient with adult-onset rod-cone dystrophy caused by CRB1 heterozygous mutations, were able to demonstrate the disease-causing potential of particular CRB1 variants in such condition (Ref. 67). Moreover, ROs derived from familial Alzheimer’s disease (AD) cell lines were proven to be significant for exploring early retinal alterations linked to AD, during which such ROs exhibited elevations in amyloid plaques, as well as phosphorylated Tau levels, characteristic of AD (Ref. 68). Others have also demonstrated the necessity of ROs as models for ocular illnesses such as glaucoma (Ref. 69), retinoblastoma (Refs 70, 71) and Stargardt disease (Ref. 72), among others. Table 1 summarizes the recent studies utilizing ROs for disease modelling, highlighting the stem cell source used in generating the ROs, the age of ROs as well as the specific gene mutations investigated.

**Table 1.** List of recent studies utilizing retinal organoids (ROs) for disease modelling

Disease model	RO age	Stem cell source	Genes investigated	Publication
Retinitis pigmentosa (RP)	Until W43 in culture	Reprogramming of RP11-derived skin fibroblasts using non-integrative RNA-based Sendai virus	RP11/PRPF31 (c.1115_1125del11 and c.522_527+10del heterozygous mutations)	(Ref. 64)
	Until W65	Reprogramming of RPGR patient-derived urinary cells to iPSCs via lentiviral or plasmid transfection	RPGR gene (mutation in exon 14 with c.1685_1686delAT, mutations in ORF15 with c.2234_2235delGA and c.2403_2404delAG)	(Ref. 63)
USH2A mutation (RP)	Until D86	Reprogramming of patient-derived urine cells to iPSCs	USH2A gene (c.8559–2A > G/c.9127_9129delTCC)	(Ref. 65)
X-linked retinitis pigmentosa	D180 and D300 RP2-knockout ROs, RP2 ROs and R120X ROs	Reprogramming and gene editing of dermal fibroblasts	R120X (nonsense mutation c.358C > T; p.R120X) and RP2 gene	(Ref. 66)
Adult-onset rod-cone dystrophy	D35 CRB1 heterozygous mutated ROs	Feeder-free culturing of patient iPSCs (LEIi006-A)	CRB1 gene (heterozygous mutations in c.1892A > G and c.2548G > A)	(Ref. 67)
Alzheimer’s disease–associated retinal alterations	Until D150	Patient-derived hPSCs obtained from arm skin fibroblasts	Familial Alzheimer’s disease gene variants (PSEN1-A246E and PSEN2-N141I)	(Ref. 68)
Glaucoma	D60	OPTN(E50K) mutation introduced to hPSCs via CRISPR/Cas9 gene editing	OPTN gene	(Ref. 69)
Retinoblastoma	Until D120	RB1 gene mutation introduced to hESCs via CRISPR/Cas9	RB1 gene (mutation of c.958C > T; p.R320X)	(Ref. 70)
	Not specified	ROs obtained from patient-derived retinoblastoma cells (RB170 cells)	MYCNOS1 gene	(Ref. 71)
	Until D105	Reprogramming of urine cells derived from a retinoblastoma patient. Compound heterozygous mutation introduced into iPSCs via CRISPR/Cas9	RB1 gene	(Ref. 73)
Stargardt disease	D185	Reprogramming of fibroblast cells derived for patients with ABCA4-associated retinopathy into iPSCs	ABCA4 gene (GRCh37 [hg19]:1:94,484,001C > T, NM_000350.3: c.5196+1137G>A)	(Ref. 72)

iPSC: induced pluripotent stem cell; hPSC/ESC: human pluripotent/embryonic stem cell.

## Organoids as potential alternatives to organ and cell transplants

Decades ago, organ transplantation had served as the sole solution for millions of people experiencing organ failures worldwide. Nevertheless, the threatening shortage of organs and the huge number of patients on waiting lists (Ref. 74), along with other obstacles, have highlighted the need to navigate potential alternatives. One of these is the ‘organoid technology’.

Numerous research publications have demonstrated the effectiveness of organoids as substitutes for organ transplants in treating various disorders, along with their ability to recapitulate in vivo organ-specific functions and architecture. It is worth noting that the transplantation of such organoids or any other human PSC-derived cells has the ability to trigger the animals’ defence systems. The most effective approach used for xenografts to circumvent this problem is the use of humanized animal models with severe immunodeficiencies. For instance, NOG (NOD/Shi-*scid IL2rγ*^null^) mice, established in Japan and mainly used for xenotransplantation studies, are characterized by decreased natural immunity with abnormal macrophage function, absence of functional B and T lymphocytes, dendritic cell dysfunctions and lack of natural killer (NK) cells, among others (Ref. 75). Furthermore, other approaches including the administration of human genes (e.g. HLA genes) and/or related growth factors, as well as the depletion of immune cells remaining in mice models of immunodeficiency via transgenic techniques utilizing herpes simplex virus thymidine kinase or diphtheria toxin A genes, have the potential to alleviate graft acceptance and hence minimize transplant rejection by hosts (Ref. 75). Other ways of immunosuppression for in vivo transplantation include administration of drugs like dexamethasone, cyclophosphamide, cyclosporine A, etc. (Ref. 76).

Exciting endeavours in the transplantation of ectodermal-derived organoids have been well documented across the literature. For instance, human BOs transplanted into the adult mouse brain exhibited long-term survival, vascularization, neuronal differential and maturation, axonal projections as well as synaptic connectivity within the host brain (Ref. 77). The transplantation of cerebral organoids in mice subjected to strokes, as well as into damaged motor cortices of rat models of TBI, aided in the repair of infarcted areas, the restoration of stroke-induced functional impairments (Ref. 78) and the alleviation of neurological motor functions (Ref. 79) respectively. Zheng and colleagues showed that on day 15, human midbrain organoids transplanted into the striatum of PD mice models exhibited axonal projections towards target regions within the host brain and enhanced motor functions, as confirmed by a number of behavioural tests including the apomorphine-induced rotation, rotarod and open-field tests (Ref. 80). Moreover, forebrain cortical organoids transplanted into the injured visual cortices of adult rats elicited neuronal efferent projections primarily towards the visual cortex and other target brain areas, received afferents from these areas and functionally integrated within the host visual circuitry (Ref. 81). In one study, human cortical organoids derived either from controls or patients suffering from Timothy syndrome (TS) were transplanted into rats’ developing cortices (Ref. 82). Interestingly, the dendritic branching pattern exhibited variations in the transplanted TS organoids when compared to controls. However, this discrepancy was not observed in TS cortical organoids grown in vitro, highlighting the importance of transplantation approaches for disease inspection (Ref. 82). These and other studies (Refs 83, 84) have revealed the capacity of transplanted BOs to structurally and functionally integrate within the host circuits.

**Figure 2.**
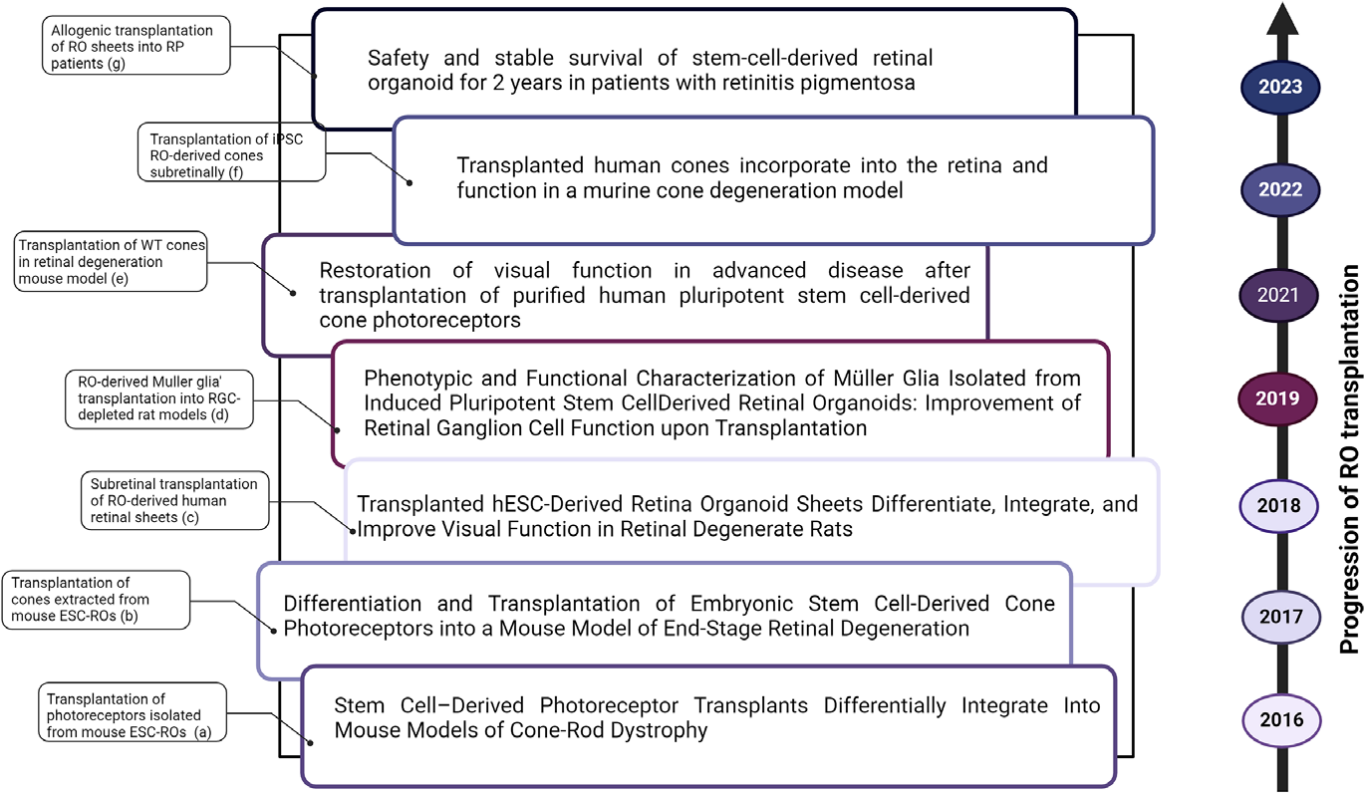
Studies highlighting the progression of retinal organoids (ROs)’ transplantation and their importance in cell replacement therapies. **A**. (Ref. 85), **B**. (Ref. 86), **C**. (Ref. 27), **D**. (Ref. 88), **E**. (Ref. 89), **F**. (Ref. 91) and **G**. (Ref. 92). Created with Biorender.

As for ROs (Figure 2), Santos-Ferreira’s team isolated photoreceptors from mouse ESC-ROs in order to assess their transplantation in mice models with either mild (prominin 1-deficient: Prom1^−/−^) or severe (cone photoreceptor function loss 1/rhodopsin-deficient double mutant: Cpfl1/Rho^−/−^) rod-cone degeneration (Ref. 85). Interestingly, the engrafted photoreceptors were shown to integrate, mature morphologically and express rod and other synaptic makers in both wild types and Prom1^−/−^ models. However, this was not the case reported for Cpfl1/Rho^−/−^, where the transplanted photoreceptors persisted within the subretinal space without morphological maturation, during which they expressed only rod specific and few synaptic markers (Ref. 85). Besides, cones extracted from mouse ESC-derived ROs were able to survive and mature within the subretinal space, once transplanted into mice models of severe retinal degeneration (Ref. 86). Additionally, mouse iPSC-derived retinal tissues transplanted into rd1 mice models of end-stage retinal degeneration were found to reconstruct rd1 outer nuclear layer during which the engrafted photoreceptors established synaptic contact with the host bipolar cells (Ref. 87). Functional assessment of the transplants was then performed using a visual behavioural test (shuttle avoidance test) that further proved light responsivity in approximately half of these mice (Ref. 87). Furthermore, McLelland and colleagues investigated the use of human ESC-RO sheets as potential therapeutics for visual restoration in rat models of severe retinal degeneration (Ref. 27). For this, retinal sheets were dissected from ROs and then transplanted into the subretinal space of immunodeficient rat models. Among their findings, the transplanted sheets underwent differentiation, integration and successfully generated functional photoreceptors and other types of retinal cells. In addition, the transplantation ameliorated visual functions in these models and demonstrated axonal projections into the host retina (Ref. 27). The transplantation of Müller glia isolated from iPSC-derived ROs enhanced RGC functions in RGC-depleted rat models, as highlighted by Eastlake’s team (Ref. 88). Ribiero’s team demonstrated the induction of light-evoked microelectroretinograms (mERGs) in retinal degeneration mice models’ (rd1/Foxn1^nu^) retinas following RO-derived wild-type (WT) cone transplant (Ref. 89). To confirm the functionality of the transplanted WT cones in rd1/Foxn1^nu^, light avoidance behavioural test was implemented during which WT mice, unlike the rd1 ones, expressed preference for the dark compartment which is expected from such nocturnal species. It was found that WT cone transplantation in rd1/Foxn1^nu^ mice shifted the preference towards the dark chamber, proving its correction-based efficacy (Ref. 89). In a study done by Zerti’s team, human ESC RO-derived cone precursors were transplanted subretinally to a mouse model of end-stage photoreceptor degeneration, receiving daily cyclosporine A injections for immunosuppression (Ref. 90). Among their findings, only 1.5% of precursors integrated within the host retina where they were able to differentiate into cone photoreceptors (Ref. 90). Despite the low engraftment yield observed, the efficacy of the procedure was further determined through functional assessment of the engrafted cells. Indeed, it was shown that half of the transplanted mice succeeded in performing visual behavioural tests, among which 33% exhibited light sensitivity in both eyes (Ref. 90). Moreover, Gasparini and colleagues transplanted iPSC RO-derived cones into the subretinal space of a cone degeneration mouse model receiving monthly triamcinolone acetonide vitrial injections to avoid risks of transplant rejection (Ref. 91). The transplanted cones were found to develop inner/outer segments, integrate deeply and extensively into the mouse outer nuclear layer without any evidence of material transfer as well as establish connections with host cells such as Müller glia and bipolar neurons (Ref. 91). Hirami and colleagues transplanted RO sheets derived from an allogeneic iPSC cell line into two patients suffering from RP (Ref. 92). Not only did the transplants survive without signs of rejection or tumorigenesis during this period but also a potential amelioration in full-field light response was reported in one of these patients (Ref. 92). In fact, this was the first clinical study to investigate RO sheet transplantation in RP humans. Further studies are required to elaborate on its efficacy and compensate for its limitations; namely, the small sample size used, the absence of a control as well as the restricted coverage provided by the transplanted retinal sheets compared to the entire retinal area (Ref. 93). In one recent study, Watanabe and colleagues investigated the efficacy of genome-edited RO (gRO) sheets in restoring visual functions once transplanted in end-stage rd1 mice, models of retinal degeneration (Ref. 94). In brief, gROs, generated via Islet 1 deletions for the purpose of reducing the number of ON bipolar cells within the grafted sheets, were utilized in particular for the transplant approach to boost host–graft interaction and avoid any competition between the graft and host bipolar cells. Among their main findings, RGCs of rd1 mice were found responsive after a 2-second flashing light following the transplantation (Ref. 94). Additionally, they were shown to be responsive under scotopic, mesopic and even photopic background conditions (Ref. 94), indicating the potential and the feasibility of such approach for visual restoration. Figure 2 highlights the key milestones of RO transplantation research beginning from animal models to the first clinical trial attempt.

In conclusion, it seems that retinal transplantation success rate might be influenced by the severity of the modelled retinal disease and the type of retinal cells being transplanted (i.e. photoreceptors/retinal sheets). More clinical trials with suitable sample sizes are needed to confirm the capacity of ROs as alternatives to organ and cell transplant approaches. In addition, a key success factor for organoid-based cell replacement therapy of retinal dystrophy resides in the ability of the transplanted photoreceptors to react to normal light stimulus. Therefore, investigating light responsivity and photostimulation in ROs is the focus of several research papers as shown in the next section.

## ROs: a focus on light responsivity and photostimulation

Various differentiation protocols have successfully generated RO models that closely resemble the primary human retina. Indeed, these organoids have demonstrated the remarkable ability to replicate the intricate five-layered structure of the adult retina with its diverse cell population (Refs 1, 95). However, a functional RO is only properly achievable if the derived mature photoreceptors are photosensitive and able to transmit the light signal to the bipolar and ganglion cells.

**Figure 3.**
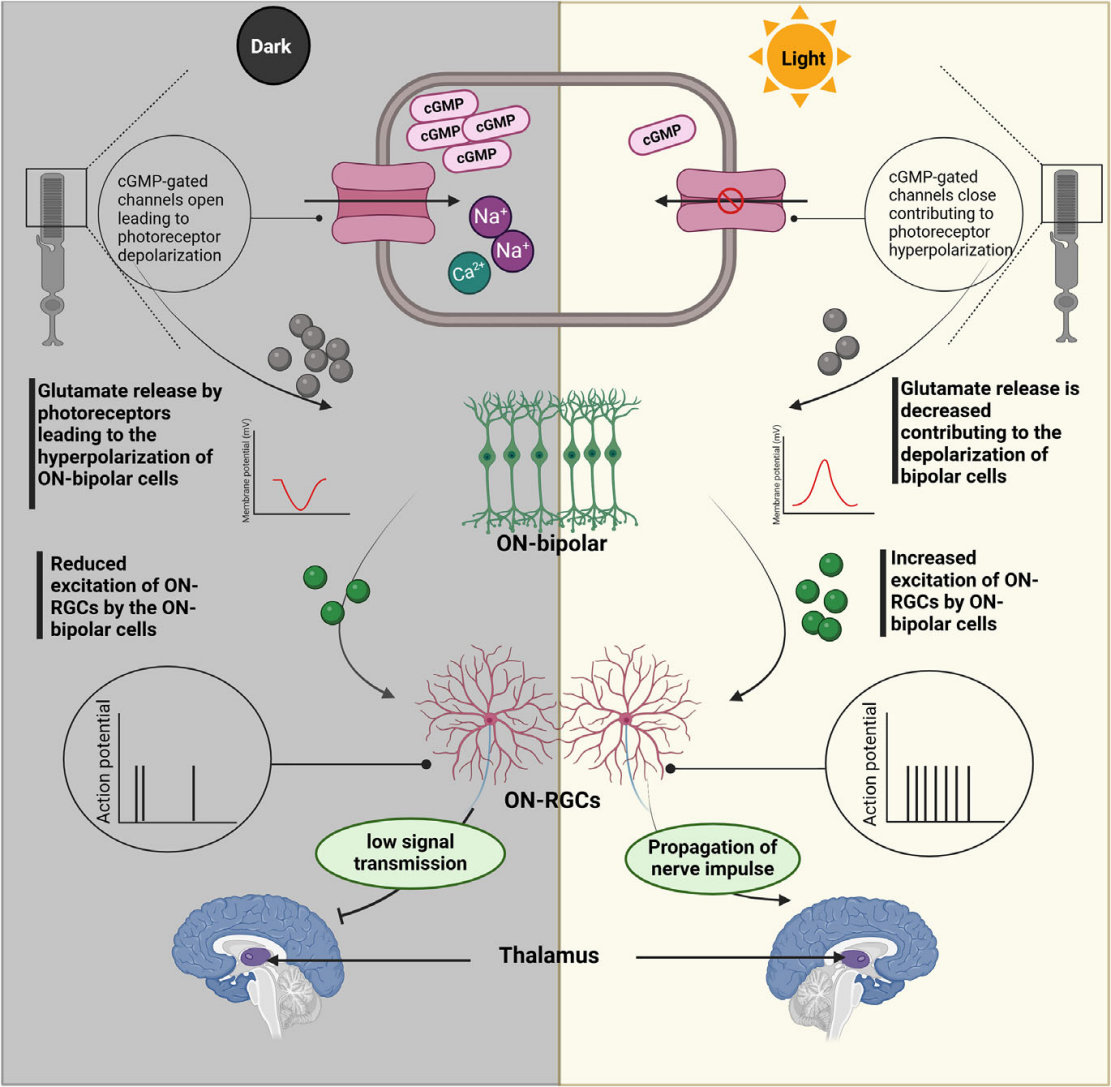
Retinal phototransduction cascade exhibited in case of dark and light conditions. In case of darkness, cGMP-gated channels open allowing the inflow of sodium (Na^+^) and calcium (Ca^2+^) cations into the outer segments. As a result, the photoreceptor cells undergo depolarization where they secrete glutamate neurotransmitters capable of inhibiting the stimulation of bipolar cells (ON type). Consequently, decreased excitation of ON-type retinal ganglionic cells (ON-RGCs) is observed leading to reduced signal transmission to central targets, namely the lateral geniculate nucleus of the thalamus (LGN). When a photon is absorbed by the photopigments, cGMP level becomes low (i.e. no opening of the corresponding gated channels) and the photoreceptors are hyperpolarized. Thus, glutamate release is decreased and the bipolar neurons are depolarized. These, in return, increase the release of the excitatory neurotransmitter onto the ON-RGCs which increase firing and become capable of propagating the nerve impulse to the LGN, along with other projection sites, for the further processing of visual information. Created with Biorender.

The process of phototransduction in the human retina is a complex mechanism. Therefore, prior to exploring the light responsivity and functional capabilities of the established ROs, first, it is essential to understand the underlying phototransduction signalling pathways occurring in the human retina in order to have insights into the expected behaviour of ROs in response to light stimuli (Figure 3). Briefly, in the human retina, in case of darkness, the cyclic guanosine monophosphate (cGMP) levels become high within the outer segment of the photoreceptor cells. Consequently, cGMP voltage-gated channels open, allowing the entry of cations such as calcium (Ca^2+^) and sodium (Na^+^), thereby creating a dark current that depolarizes the photoreceptors. Once depolarized, the photoreceptors release glutamate neurotransmitter, which stimulates the metabotropic glutamate receptors on the bipolar cells causing an inhibitory effect (i.e. ON bipolar cells hyperpolarized). This causes a decrease in neurotransmitter release from the ON bipolar cells reducing therefore their ability to excite ON retinal ganglionic cells (ON-RGCs), which serve as the sole connection between the retina and the brain (Ref. 96). Conversely, in the presence of light, cGMP voltage-gated channels close, preventing the influx of Na^+^ and Ca^2+^ into the outer segment. In addition, an efflux of potassium cations (K^+^) from the inner segment is observed, altogether leading to photoreceptor hyperpolarization. In this case, glutamate release is decreased leading to the depolarization of ON bipolar cells and increase in their neurotransmitter release, provoking subsequently an increase in the firing of ON-RGCs. Hence, RGCs become capable of propagating the increased nerve impulses to central targets, mainly the lateral geniculate nucleus (LGN) of the thalamus responsible for the processing of visual information, as well as to many other projection sites (Ref. 97) (Figure 3). An opposite mechanism simultaneously occurs for OFF bipolar cells having ionotropic glutamate receptors (Ref. 98).

These interactions and synaptic connectivity existing among the different cell types populating the distinct retinal layers form the foundation of the retinal phototransduction cascade. As a matter of fact, the efficiency of ROs in achieving a mature state with a multi-layered configuration gives hope in their ability to react to light stimuli, regardless of some challenges encountered like RGC loss in long-term cultures (Ref. 99).

Indeed, several studies throughout the literature have addressed the photoresponsivity of ROs using various methodologies (Table 2). For instance, in a study conducted by Zhong’s team, photoreceptors of week 25 human iPSC-derived retinal cups (W25 hiPSC-derived RC) expressed several proteins implicated in rod phototransduction cascade (Ref. 99). Furthermore, they assessed the receptors’ photoresponsivity using perforated patch-clamp method on rod cells in W25–W27 hiPSC-derived RCs. Interestingly, only 2 of the 13 randomly chosen cells showed response to light, knowing that their outer disc segments were just beginning to form (Ref. 99). Hallam and colleagues also investigated light responsivity of different iPSC-derived ROs using multielectrode array (MEA) recordings (Ref. 100). In fact, upon exposure to high-intensity light stimulus, either an increase or decrease in spiking activity was recorded from presumable RGCs that appeared to be similar to those observed in the developing retina (Ref. 100). Furthermore, the latter ROs at day 150 of differentiation were found responsive to puffs of GABA and 8-br-cGMP (cGMP analogue), excluding any effect of intrinsically photosensitive RGCs on the obtained recordings (Ref. 100). Besides, MEA recordings from RGCs showed a 25% increase in spiking activity upon exposure to a strong white light pulse stimulus (Ref. 101). In another study utilizing MEA, a 25% increase and a 25% decrease in spiking activities were reported from ON-RGCs and OFF-RGCs, respectively, following exposure to a high-intensity light stimulus (Ref. 102) where 8-br-cGMP puff was then used to discriminate between intrinsically photosensitive RGCs and photoreceptor-driven ones. Moreover, Cowan and colleagues investigated the functionality of synapses in mediating light transmission within ROs using calcium imaging (Ref. 95), during which 39 of 233 photoreceptors responded to light stimulus. Glutamatergic synaptic transmission blockade did not halt the latter light responses, confirming the attribution of the signals to these light-sensitive photoreceptors (Ref. 95). In another study, over 100 cones from stage 3 ROs (stage 3: hair-like photoreceptor outer segments develop on the surface of ROs) were assessed for light responsivity (Ref. 103). It was shown that approximately 35% of these cones elicited light-evoked voltage responses, similar to the ones exhibited by macaque foveal cones (Ref. 103). In a study conducted by Celiker et. al., ROs derived from human PSCs were developed for the further investigation of retinal light-responsive mi-RNAs (Ref. 104). To test the light responsivity of these organoids, MEAs were utilized where ON-RGCs and OFF-RGCs responded with increased and decreased spiking activities, respectively, upon light stimulus. An analogue of cGMP, 8-br-cGMP, was then used to mimic the dark current in order to make sure that the latter responses were caused by phototransduction and not just due to intrinsic photosensitivity of RGCs. Indeed, upon 8-br-cGMP puff, the spiking activity was reduced for ON-RGCs, while it was elevated for the OFF cells, indicating the presence of a phototransduction cascade (Ref. 104). Interestingly, they also showed that ROs at day 120 or more exhibit photoresponsivity at the miRNA transcriptional level (Ref. 104) (see Table 2).

**Table 2.** Summary of the main studies investigating light-evoked responses in retinal organoid (RO) models

Model	Stimulus	Light responsivity assessment	References
Weeks 25–27 hiPSC-derived retinal cups	Light flashes	Perforated patch clamping	(Ref. 99)
WT- and AMD iPSC-derived ROs	High-intensity light stimulus (200 ms, 217 μW/cm^2^ irradiance, 1 Hz)	BioCam4096 MEA	(Ref. 100)
hiPSC- and hESC-derived ROs	White light pulse (200 ms, 217 μW/cm^2^ irradiance, 1 Hz)	BioCam4096 MEA	(Ref. 101)
hiPSC-derived ROs	Light	Calcium imaging/two-photon laser imaging	(Ref. 95)
Stage 3 ROs	LED light flashes (wavelengths of 410, 505 and 650 nm)	Voltage and patch-clamp recordings	(Ref. 103)
hPSC-derived ROs	Photostimulation (LED)	MEA	(Ref. 104)

WT: wild type, AMD: age-related macular degeneration, hiPSC/ESC: human-induced/embryonic stem cells, MEA: multielectrode array.

In conclusion, light-responsive RO models can be successfully generated by ensuring the fulfilment of all conditions required for an effective signal transmission such as a suitable membrane conductivity of photoreceptors, the establishment of synaptic connectivity and the proper functioning of the phototransduction cascade (Ref. 105). A hindering problem in achieving these conditions resides in the lack of vascularization and hence the lack of the necessary nutrients required for long-term in vitro cell survival. Therefore, researchers investigated new solutions such as microfluids and organ-on-chip systems described in the next section.

## Approaching BOs and ROs through microfluidics

The integration of microfluidic systems and on-a-chip devices with diverse organoid types has led to significant enhancements in these models (Ref. 106). In fact, microfluidic systems are based on incorporating microchannels capable of transporting specific fluids into either organs or organoids to ensure their interaction with the surrounding environment. Despite successful approaches in organoid establishment, several drawbacks have been shown to be alleviated through microfluidics or ‘organ on-a-chip’ devices. The lack of vasculature in organoids, which hinders their ability to fully recapitulate in vivo systems, is regarded as one of the major limitations encountered in the current models (Ref. 107).

For instance, in a study done by Abdulla’s team, cerebral organoids incorporated into a microfluidic system showed no inner core cell death for a total period of 50 days, along with improved cellular proliferation and maturation as confirmed by immunostainings, proteomics and metabolomics (Ref. 108). Furthermore, a modified form of the latter microfluidic system enabled them to further investigate the neurotoxic effects of bisphenol S, a synthetic compound used in our everyday life, on brain development (Ref. 109). Moreover, a 3D-printed microfluidic device enabled the generation of more uniform, adequately structured cerebral organoids that were maintained without any manual intervention for at least 10 days, thereby reducing handling time and hence contamination chances with long-term cultures (Ref. 110). Besides, BOs integrated into a continuous stirred-tank reactor (CSTR)-inspired mesofluidic bioreactor platform offering continuous nutrient supply, exhibited reduced batch-to-batch variability, as well as decreased cell death, among others (Ref. 111).

In fact, the use of this approach with ROs is considered quite novel. The concept of microfluidics in combination with ROs was introduced following the realization that the latter organoids lack the adequate physical microenvironment required for boosting their performance.

A number of research publications have demonstrated the necessity of incorporating microfluidic systems for the generation of more enhanced RO models. For instance, Achberger and colleagues developed a retina-on-a-chip (RoC) model by integrating ROs and retinal pigmented epithelium (RPE) within a microfluidic system (Ref. 112). In brief, RPEs and ROs were cultured in tissue chambers of a microfluidic apparatus, where they were continuously supplied by specific factors delivered through an underlying microchannel for a vasculature-like perfusion (Ref. 112). Among their findings, RoC accelerated the development of outer segment-like structures compared to RPE-deficient RO chips and those cultured on ordinary dishes. Visual-related functions, namely outer segment phagocytosis and calcium dynamics, were also reported in such platforms (Ref. 112). A controllable perfusion microfluidic chip (CPMC) was introduced by Gong’s team to enhance RO culture conditions (Ref. 113). Indeed, it was shown that ROs perfused under this platform exhibited enhanced neural retina (NR) induction with more thickened NR structures being prevalent, increased retinal progenitor cell proliferation and improved RGC development. Furthermore, the activated voltage-gated channels, along with the elevated extracellular matrix component (ECM) expression, facilitated RO development as well as RGC differentiation in this system (Ref. 113). Moreover, bioreactor-grown ROs exhibited increased number of photoreceptors, enhanced stratification of the retinal layers, decreased cell death and increased cell proliferation (Ref. 114). In a study done by Amos and colleagues, a two-chamber polydimethylsiloxane-based microfluidic device with axon guidance channels was developed during which retinal spheroids incorporated within this system projected towards thalamic targets through up to 6-mm-long channels, modelling the retinogeniculate pathway (Ref. 115).

Microfluidic chip approaches in combination with ROs have been also regarded as indispensable for the investigation and recapitulation of ocular illnesses such as RP (Ref. 116). In this study, ROs and RPEs derived from patients with USH2A mutations were utilized for RP disease modelling. In fact, USH2A mutation contributed to the anomalous development of RPEs, ROs’ increased apoptosis, as well as aberrant ECM organization functions within ROs (Ref. 116). Nevertheless, the implementation of a microfluidic chip for culturing proved to be advantageous as it enhanced the synthesis of ECM components by the diseased ROs and further stimulated the development of ROs and RPEs in comparison to traditional culture conditions (Ref. 116). Additionally, retinal chips have been utilized to simulate blood–retina barriers (BRBs), including the outer BRB made up of RPEs with the adjacent choroidal microvasculature and the inner BRB where neural retina and a vascular network become integrated (Ref. 117).

In conclusion, the incorporation of ROs into microfluidic systems appears to facilitate a deeper comprehension of human retina development, thereby improving the recapitulation of in vivo organ characteristics in both normal and pathological states. Further investigation of ROs’ light stimulation and responses within microfluidics is required as no studies so far assessed such aspects.

## Shortcomings and challenges of organoids

Several challenges were encountered with the use of organoid systems. Among these, the lack of regional interaction poses a significant obstacle, especially when it comes to disease modelling. A particular illness affecting an in vivo organ tends to be influenced by the surrounding areas and other regions of interaction. The absence of this aspect, thereby, hinders the organoids’ ability to accurately mimic complex disease processes and pathways. Secondly, the lack of a perfused vasculature presents a notable limitation. The primary role of vascularization is the transportation of oxygen, nutrients, metabolic wastes, etc. The fact that the currently available organoid models are devoid of vascularized networks amplifies their chances of developing necrotic cores (Ref. 118), potentially reducing their lifespan. Furthermore, the variability between batches (Ref. 119) and among organoids (Ref. 120) raises concerns about the reproducibility of these models. Such variability should be addressed, as it can impede the ability to generalize research findings. Other limitations include cellular stress, hypoxia and necrosis, restricted maturation and complex topographic organization (Ref. 32).

## Assembloids: a turning point in disease modelling and axonal pathfinding investigation

As mentioned earlier, the lack of regional interaction in the current organoid models has led to concerns, raising questions about their feasibility in reflecting in vivo systems. This explains why numerous research works have been directed to compensate for this issue by aiming to develop more enhanced representative in vitro structures that not only can combine multiple regions/organoids into a single model but also can incorporate missing cell populations within individual organoids. These 3D-assembled structures are referred to as assembloids. Assembloids, as previously highlighted by Sergio Pasca, are fusion structures that can combine multiple organoid types for the purpose of accounting for inter-regional interactions or can even incorporate missing cell types within the same organoids (Ref. 34).

To date, different types of assembloids have been generated for the further investigation of regional interactions. Table 3 provides a summary of brain and retinal assembloids that have been generated up to date.

**Table 3.** Summary of diverse retinal and brain assembloid models that have been recently established

Assembloid type	Development mode	Reference
Ventral midbrain–striatum–cortex assembloids	Linear fusion of individual ventral midbrain, striatal and cortical organoids using polydimethylsiloxane (PDMS) embedding moulds	(Ref. 121)
Cortical–blood vessel assembloids	Fusion of cortical and blood vessel organoids once placed in close proximity	(Ref. 122)
Vascularized brain assembloids	Organoid dissociation, aggregate formation and addition of common myeloid progenitors (CMPs) for assembloid development	(Ref. 123)
Cortico-striatal assembloids	Fusion of human cortical and striatal spheroids when placed in close proximity	(Ref. 124)
Midbrain–striatal assembloid	Co-culture of midbrain and striatal organoids obtained from the respective precursor spheroids	(Ref. 125)
Retino-cortical assembloids	Retinal and cortical organoids situated in close proximity	(Ref. 126)
Assembled model of human pluripotent stem cell-derived organoids	Fusion of retinal, thalamic and cortical organoids on membranes for more controlled positioning	(Ref. 127)

The lack of vascularization in most of the generated organoid models poses a significant challenge in mimicking in vivo microenvironments. The incorporation of vascular networks into assembloids offers a potential solution, enabling better recapitulation of in vivo systems, as well as disease pathophysiology. For instance, cortical–blood vessel assembloids obtained upon fusing cortical and blood vessel organoids were shown to be more effective in outlining SARS-COV-2 pathology, compared to BOs (Ref. 122). Additionally, vascularized brain assembloids showed accelerated maturation of astrocytes, high numbers of synapses, as well as improved proliferation of neuroepithelial cells when compared to organoids (Ref. 123). Besides, elevated levels of total tau and phosphorylated tau, among others, were reported in the following assembloids harbouring the tau^301S^ mutation, highlighting their significance in the study of tauopathies (Ref. 123).

The investigation of axonal projection, as well as inter-regional communication, was also facilitated via assembloid employment. Indeed, in a study conducted by Miura et. al., cortical–striatal assembloids, obtained upon fusing human cortical spheroids (hCSs) with human striatal spheroids (hStrSs), showed unilateral axonal projections from hCSs towards hStrSs (Ref. 124). In addition, the projecting hCS neurons were found to approach postsynaptic dendritic protein PSD-95 expressed on the dendrites of hStrS neurons (Ref. 124). Furthermore, the linear fusion of ventral midbrain, striatal and cortical organoids resulted in the establishment of an assembloid model exhibiting dopaminergic neuronal projection from the ventral midbrain towards striatal and cortical targets (Ref. 121). Moreover, a dual organoid platform consisting of an inter-organoid pathway (IOP) linking midbrain and striatal organoids at opposing ends was successfully generated (Ref. 125). Interestingly, green fluorescent protein (GFP)-labelled midbrain organoids’ neurites were found to project deep into the striatal ones, highlighting efficient synaptic formation within IOPs (Ref. 125). Others have demonstrated the capacity of 3D assembloids to recapitulate neuronal descending pathways necessary for muscle activation and movement (Ref. 128), as well as ascending sensory pathways implicated in the processing of somatosensory information such as pain and itch (Ref. 129).

Fernando and colleagues demonstrated the ability of retinal and cortical organoids, once placed in close proximity, to connect through optically derived axonal projections (Ref. 126). Interestingly, retinal MAP2-RGC axons propagated into the BOs, forming nerve bundle-like structures (Fernando et al., 2022). Furthermore, THY1 (THY1: RGC marker)-positive RGC axons in the centre of the ROs were found to project into BOs, specifically towards THY-1/TBR1 (cortical marker)-positive neurons (Ref. 126). Only one study conducted by Fligor’s team revealed the possibility of combining retinal, thalamic and cortical organoids into a single assembloid fusion structure (Ref. 127). This study was one of its kind in addressing the issue of RGC loss in long-term cultures, especially after being able to demonstrate their maintenance and enhanced survival in long-term assembloids. Indeed, RGC axons’ extension towards CTIP2-positive BOs within retino-cortical assembloids was observed (Ref. 127). As a matter of fact, RGC survival within ROs was enhanced when the latter was grown as part of an assembloid, 7 days postassembly. Moreover, retinotectal projections were then examined using a fusion model combining retinal, thalamic and cortical organoids into a single tri-assembloid structure (Ref. 127). Among their findings, tdTomato-expressing RGCs projected their axons into the GFP-expressing thalamic organoids which, in return, extended their corresponding neurites towards CTIP2-positive cortical organoids (Ref. 127). These studies, indeed, provide insights into the accuracy and reliability of assembloid models in mirroring axonal pathways.

Numerous assembloid types have been reported across the literature including the brain and retinal fusion models. Nevertheless, the implementation of retinal assembloids for the investigation of axonal projection and pathfinding, in particular, appears to be quite novel, as it has only been recently initiated. One peculiar approach that might pave the way for the future of generating more profound 3D model systems is that of air–liquid interface (ALI) culture. Indeed, cerebral organoids developed through ALI exhibited improved morphology, as well as enhanced survival, when compared to whole organoids (Ref. 130). Furthermore, specifically oriented axonal bundles that were reinforced with time were observed in ALI–cerebral organoids. Additionally, microelectrode array (MEA) recordings of the ALI cultures revealed the functional connectivity of neural networks (Ref. 130). To our knowledge, no study has proceeded with the establishment of retinal assembloids using ALI.

## Conclusion and future perspectives

The breakthrough in stem cell research and subsequently in the field of organoid technology offered profound insights and allowed for a deeper comprehension of in vivo complex functions. Not only has this milestone expanded possibilities for mimicking in vivo systems but it also facilitated disease simulation, drug screening as well as personalized medicine. 3D organoids are unparalleled human in vitro models recapitulating various disease pathophysiologies and complementing therefore existing knowledge revealed by traditional 2D cultures and animal models.

In the past few years, great progress has been made in organoid research, bringing hope for understanding disease onset and finding proper treatment. However, there are still several issues in organoid culture that still need to be addressed in order to be able to use them on large scale for cell replacement therapies and drug screening. These issues include the long and cumbersome cultivation protocols, poor reproducibility and sustainability due to the batch-to-batch variation, lack of vascularization within the organoids as well as ethical issues regarding stem cell sources.

These challenges are in focus of the current research which implemented new cutting-edge technologies such as microfluidic devices, organ on chip or air–liquid interface, as well as more complex 3D structures combining and fusing several region-specific organoids, namely assembloids, or integrating other cell types in a chimaera organoid. Together, these technologies could offer more powerful and accurate human-relevant models for various applications. Therefore, further research and exploration are still needed, and only the future will reveal the promising potential of these tools.
